# Subthalamic Neural Activity Patterns Anticipate Economic Risk Decisions in Gambling

**DOI:** 10.1523/ENEURO.0366-17.2017

**Published:** 2018-02-06

**Authors:** A. Mazzoni, M. Rosa, J. Carpaneto, L. M. Romito, A. Priori, S. Micera

**Affiliations:** 1 Translational Neural Engineering Area, The Biorobotics Institute, Scuola Superiore Sant’Anna, Pontedera, 56025, Italy; 2 Clinical Center for Neurostimulation, Neurotechnology and Movement Disorders Fondazione IRCCS Ca’ Granda Ospedale Maggiore Policlinico, Milan, 20122, Italy; 3 Movement Disorders Department, Neurological Institute Carlo Besta, Milan, 20133, Italy; 4 ”Aldo Ravelli” Research Center for Neurotechnology and Experimental Brain Therapeutics, Department of Health Sciences, University of Milan Medical School, Milan, Italy; 5 Bertarelli Foundation Chair in Translational NeuroEngineering, Institute of Bioengineering and Center for Neuroprosthetics, School of Engineering, École polytechnique fédérale de Lausanne, Lausanne, CH-1015, Switzerland

**Keywords:** Gambling disorder, local field potentials, Parkinson’s disease, subthalamic nucleus

## Abstract

Economic decision-making is disrupted in individuals with gambling disorder, an addictive behavior observed in Parkinson’s disease (PD) patients receiving dopaminergic therapy. The subthalamic nucleus (STN) is involved in the inhibition of impulsive behaviors; however, its role in impulse control disorders and addiction is still unclear. Here, we recorded STN local field potentials (LFPs) in PD patients with and without gambling disorder during an economic decision-making task. Reaction times analysis showed that for all patients, the decision whether to risk preceded task onset. We compared then for both groups the STN LFP preceding high- and low-risk economic decisions. We found that risk avoidance in gamblers correlated with larger STN LFP low-frequency (<12-Hz) fluctuations preceding task onset. In particular, the amplitude of low-frequency LFP fluctuations carried significant information about future decisions. Decisions of patients not affected by gambling disorder were instead not correlated with pretask STN LFP. Our results suggest that STN activity preceding task onset affects risk decisions by preemptively inhibiting attraction to high but unlikely rewards in favor of a long-term payoff.

## Significance Statement

Economic decision-making relies on a balance between impulsiveness and rationality, which is disrupted in individuals with gambling disorder. Parkinson’s disease (PD) patients receiving dopaminergic therapy are at higher risk of developing this disorder. Here, we compared the neural activity recorded in the subthalamic nucleus of PD patients with and without gambling disorder during an economic decision-making task. We found that neural activity in this area is different in gamblers and that is possible to estimate gamblers’ attitude toward risk on single bets based on the observed low-frequency extracellular fluctuations. These findings will help clarify the role of the subthalamic nucleus in decision-making and pave the way to PD therapies with a lesser risk of cognitive side effects.

## Introduction

Humans make fast and efficient decisions even when the outcomes associated with each option are probabilistic, as is often the case in real life. Economic decision-making can be impaired in psychiatric or neurologic pathologic conditions, such as gambling disorder (GD), a problematic addictive behavior (American Psychiatric Association, 2013) with a particularly high incidence in Parkinson’s disease (PD) patients receiving dopamine replacement therapy (∼5% vs. ∼1% over the whole population; Santangelo et al., 2013; Weintraub et al., 2015). Understanding the psycho-pathophysiological mechanisms of GD in PD patients would improve PD and GD therapies and further inform the neural basis of economic decision-making. PD patients with GD (GDPs) are more likely than PD patients without GD (NGDPs) to follow the impulse of betting despite the negative consequences of such action. However, their behavior is nondeterministic, as they resist their propensity to risk a significant fraction of times that the option of a high-risk choice is presented. Human behavior is known to strongly depend on internal bias (Kahneman and Tversky, 1979) that can often be associated with specific neural features (De Martino, 2006; Sacré et al., 2016). What are then the neural correlates of the trial-to-trial variations of the attitude toward risk in GDPs? In particular, what happens when GDPs manage to overcome their general behavioral tendency and avoid risk?

We investigated the hypothesis that subthalamic nucleus (STN) activity reflects the internal state determining the attitude toward risk on a single-trial basis, given the wealth of data indicating an involvement of this region in decision-making. Studies about stop-signal tasks (Ray et al., 2012; Alegre et al., 2013) and high-conflict tasks (Frank et al., 2007; Brittain et al., 2012) have shown that the STN is involved in reactive inhibition (i.e., behavioral inhibition triggered by the STN activity after stimulus presentation; Aron, 2011; Jahanshahi et al., 2015a, b). The STN is also involved in proactive inhibition (Aron, 2011; Jahanshahi et al., 2015b), since the STN activity preceding stimulus presentation leads to inhibition of upcoming impulses to initiate a movement (Favre et al., 2013; Benis et al., 2014; Obeso et al., 2014). The STN inhibitory role is not limited to motor control, but extends to impulse control in cognition and emotion (Jahanshahi et al., 2015b); however, its role in GD and other impulse control disorders is still unclear (Jahanshahi et al., 2015a; Zavala et al., 2015). Electrodes implanted in the STN for deep brain stimulation (DBS) in PD patients have been used to investigate correlations between decision-making and spike rates (Zaghloul et al., 2012) and low-frequency local field potentials (LFP) in the STN (Cavanagh et al., 2011; Herz et al., 2016; Zénon et al., 2016). Crucially, it has been shown that STN LFPs differ between GDPs and NGDPs in the following conditions: (a) at rest (Rodriguez-Oroz et al., 2011), (b) while making a choice between two known options (Rosa et al., 2013), and (c) when evaluating the consequences of a choice (Fumagalli et al., 2015). However, STN activity preceding options presentation has never been analyzed to assess the correlation between STN and risk propensity in GDPs and/or NGPDs.

To clarify this relationship, we compared the behavior and the STN LFP of GDPs and NGDPs choosing between high-risk (HR) and low-risk (LR) economic options (see Methods). We found no correlation between STN LFP and NGDPs risk attitude. GDPs risk attitude was instead determined before options presentation, and the low-frequency (<12-Hz) component of STN LFP within that interval significantly correlated with future decisions.

## Materials and Methods

### Experimental design

#### Patients, clinical data analysis, and neurosurgical procedures

The LFP study involved 12 patients with advanced PD, already scheduled for a subthalamic implant to treat their motor symptomatology. All the patients provided written informed consent for STN DBS or LFP study. The study was approved by the institutional review board and conformed to the Declaration of Helsinki.

Complete analysis of patients’ clinical data, details of neurosurgical procedures, LFP signal preprocessing, and economic task design are described below and in Tables 1 and 2. Briefly, enrolled patients were classified as patients with gambling disorder (GDPs) or without it (NGDPs) according to DSM-5 diagnostic criteria (American Psychiatric Association, 2013); gambling history was ascertained during a structured psychiatric and behavioral interview, and gambling behavior was scored by using the South Oaks Gambling Screen (SOGS; Lesieur and Blume, 1987). Groups were formed first selecting six GDP volunteers and then forming a matching group of six NGDPs.

**Table 1.** Patients’ demographic and clinical characteristics

				Preoperative therapy							
Patient	Age at implant (years)	Gender	Disease duration at implant (years)	LEDD (mg)	DA in LEDD (mg)	Preoperative UPDRS III score	MMSE	SOGS	BIS	Y1-STAI	Y2-STAI
Cohort 1: GDPs											
1	49	M	8	1400	0	73.9	26.6	7	18	29	41
2	41	M	15	550	0	73.5	24.6	9	7	20	34
3	52	M	4	200	0	50.0	27.1	10	24	35	28
4	60	F	6	940	240	61.3	27.5	5	26	55	53
5	78	F	10	1200	280	56.2	30	7	19	46	35
6	48	M	10	900	0	71.1	27.6	15	22	48	36
Mean (SD)	53.0 (10.0)		8.8 (3.8)	865.0 (435.0)	86.6 (134.5)	64.3 (10.0)	27.2 (1.7)	8.8 (3.5)	19.3 (6.7)	38.8 (13.1)	37.8 (8.5)
Cohort 2: NGDPs											
1	67	F	14	2275	420	38.2	28.49	0	26	41	46
2	63	F	9	2575	70	62.9	26.27	0	22	22	38
3	60	F	14	1550	350	70.3	28.27	1	25	52	54
4	64	F	11	910	0	61.5	29.27	0	22	33	39
5	47	M	10	820	240	58.0	25.89	0	14	38	31
6	61	F	11	1100	210	60.7	NA	0	NA	39	47
Mean (SD)	60.0 (6.0)		11.5 (2.1)	1152.1 (691.5)	215 (160)	58.6 (10.8)	27.6 (1.5)	0.2 (0.4)	21.8 (4.7)	37.5 (9.8)	42.5 (8.1)
*p*-value	*p* > 0.05	*p* > 0.05	*p* > 0.05	*p* > 0.05	*p* > 0.05	*p* > 0.05	*p* > 0.05	*p* ≤ 0.05	*p* > 0.05	*p* > 0.05	*p* > 0.05

BIS, Barratt Impulsiveness Scale; DA, dopamine agonist; LEED, Levodopa Equivalent daily dose; MMSE, Mini-Mental State Examination; NA, not available; SOGS, South Oaks Gambling Screen; UPDRS III, Unified Disease Rating – scale motor score; Y1–Y2 STAI, State–Trait Anxiety Inventory. Patients with gambling disorders (GDs) and without (NGDs) were compared with a paired *t* test for age and disease duration at implant, preoperative therapy, preoperative UPDRS motor score, preoperative MMSE, SOGS, BIS, and Y2 STAI, stereotactic coordinates; Fisher’s exact test for gender. Differences were considered significant if *p* < 0.05. Data were expressed as mean ± SD.

**Figure 1.**
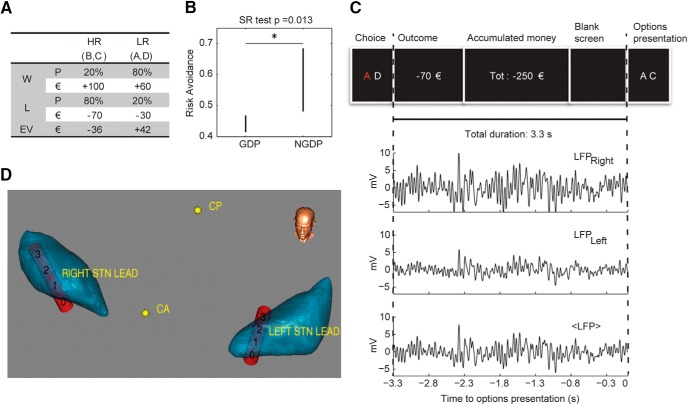
Economic task and STN LFP recordings. ***A***, Letters, wins (W) and loss (L) probability (P) and value (€), and expected values (EV) associated with low-risk (LR) and high-risk (HR) options. ***B***, Risk avoidance in GDPs (*n* = 6) and NGDPs (*n* = 6). Bars represent median confidence interval. Marker indicates significant difference (*p* = 0.013). ***C***, Window of interest (3.3 s preceding options presentation). Top, sequence of visual stimuli in the window of interest. Bottom, processing of recorded LFP: raw data from right and left leads, and average LFP. ***D***, 3D reconstruction of STN location of the STN (blue structures) and of the Medtronic 3389 DBS leads (red cylinders) with 0–3 contacts in one example GDP subject (#6). CA and CP indicate the anterior and posterior commissure.

All patients underwent a one-stage bilateral stereotactic subthalamic implant, according to standard procedures (Zangaglia et al., 2009; Franzini et al., 2012). During the economic task, LFPs were simultaneously captured from the contact pair 0-2 of the DBS electrodes (Fig. 1*D*). We also enrolled 17 healthy subjects comparable to patients in age and education. These subjects performed exactly the same two-alternative forced-choice task as PD patients for behavior comparison.

We collected clinical data such as gender, age, disease duration, disease onset, preoperative therapy [levodopa equivalent daily dose (LEDD) and dopamine agonist dosage in LEDD], preoperative score on the motor part of the Unified Parkinson’s Disease Rating Scale (UPDRS III), off and on medication. All patients underwent a complete cognitive and psychological evaluation, including Mini-Mental State Examination (MMSE; Folstein et al., 1975) and State-Trait Anxiety Inventory (STAI; Spielberger et al., 1983) to exclude cognitive, mood, and anxiety disorders. Clinical data are reported in Table 1; GDP and NGDP groups were comparable for demographic and PD characteristics, except for a significant difference in SOGS score (Table 1, bottom row). The final set size was 6 for each group. This number was sufficient to perform descriptive statistics and within-group significant (*p* < 0.05) paired Wilcoxon test between conditions. However, results on reaction time statistics, LFP fluctuations comparison, and information measurements were computed normalizing the variables subjectwise and then pooling trials within all subjects on the same group to increase the robustness of the results (see Dataset limitations).

**Figure 2.**
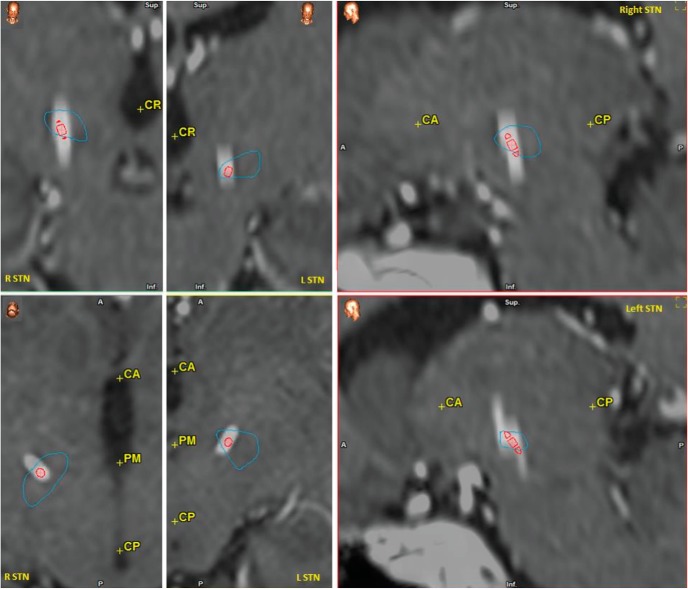
2D reconstruction of recording coordinates. Location of STN (pale blue lines) and of Medtronic 3389 DBS contact 1 in GDP #6 (already shown in Fig. 1*D*). R, right; L, left; CA, anterior commissure; CP, posterior commissure; PM, middle point between CA and CP line; CO, MRI coronal view; SW, MRI sagittal view; AX, MRI axial (transverse) view; Sup, superior; Inf, inferior; A, anterior; P, posterior.

All patients underwent a one-stage bilateral stereotactic subthalamic implant, according to standard procedures (Zangaglia et al., 2009; Franzini et al., 2012). Briefly, initial STN coordinates were determined by matching the patient’s preoperative brain CT and MRI fused images with a digitized stereotactic atlas. Combined electrodes for both intraoperative recording and macrostimulation were then used to check and choose the correct location of the definitive STN lead. Each implanted lead (DBS Lead Model 3389, Medtronic) has four cylindrical contacts (1.27-mm diameter, 1.5-mm length, placed 2 mm apart, center-to-center) denominated 0-1-2-3, beginning from the ventral contact. After implant, the extracranial section of the STN lead was connected to an externalized extension wire to permit the LFP recordings. A complete 2D and 3D reconstruction of STN lead location was ascertained by combining the findings of the Medtronic Stealth Station TREON plus Navigation System with the findings of Medtronic Optivise software: 3D anatomy of basal ganglia was adapted to the brain geometry of each patient by overlaying the preoperative and postoperative MRI or CT scans onto the software atlas. STN leads were considered correctly positioned only if two or more contacts included the STN. A 2D reconstruction of STN lead contacts 1 location is provided in Fig. 2 (referred to GDP #6), and a 3D reconstruction of STN leads location (also referred to GDP #6) is provided in Fig. 1*D*. Stereotactic coordinates for all subjects are reported in Table 2. After the end of LFP recording, the STN leads were connected by tunneled extension to the implantable pulse generators (Activa PC Neurostimulator Model 37601 or Activa SC Neurostimulator Model 37603, Medtronic), placed in a subclavicular subcutaneous pouch.

**Table 2.** STN lead contact 1 stereotactic coordinates

	Contact 1 stereotactic coordinates	
Patient	Right (*x*, *y*, *z*)	Left (*x*, *y*, *z*)
Cohort 1: GDPs		
1	12.4, –4.7, –0.3	11.3, –6.1, –4.7
2	11.2, –3.4, –3,1	11.4, 6.6, –6.7
3	13.0, 1.3, –3.0	10.3, –0.3, –3.3
4	11.6, –3.0, –1.5	10.3, –4.1, –1.6
5	10.9, –1.4, –3.1	11.9, –2.9, –5.5
6	13.1, –1.3, –3.6	9.9, 0.3, –6.9
Mean (SD)	12.0 (0.9), –2 (2), –2.4 (1.2)	10.8 (0.8), –1.1 (4.4), –4.8 (2)
Cohort 2: NGDPs		
1	9.8, 4.2, 0.75	11.8, –6.2, –1.8
2	11.7, –3.4, –4.2	11.8, –3.2, –4.0
3	9.6, –1.9, –5.1	10.3, –4.4, –5.0
4	10.7, –1.2, –2.6	11.3, –3.1, –2.1
5	12.4, –2.8, –4.3	12.1, –4.0, –3.7
6	13.6, –4.5, –3.8	11.3, –3.0, –4.0
Mean (SD)	11.3 (1.6), –1.6 (3), –3.2 (1.2)	11.4 (0.6), –3.9 (1.2), –3.4 (1.2)
*p*-value	*p* > 0.05	*p* > 0.05

Stereotactic coordinates of subthalamic leads’ contact 1 were rendered according to the anterior commissural–posterior commissural (AC–PC) line and the mid-commissural point (MCP) between AC-PC line; *x* = mm lateral from AC-PC line; *y* = mm anterior (+) or posterior (–) from MCP; *z* = mm depth according to AC–PC line (–, if ventral; +, if dorsal). Differences were considered significant if *p* < 0.05. Data were expressed as mean ± SD.

#### Economic decision-making task

Participants were seated in front of a computer screen in a lighted room. All patients were studied in the “on levodopa” condition. Pairs of stimuli (two of the four letters: A, B, C, and D) were presented on the screen in white on a black background (Fig. 1*C*). We call “trial” each options presentation followed by a choice, and “session” the set of the trials for each subject. Subjects were asked to choose a stimulus by pressing one of the two keyboard keys, corresponding to the stimulus on the left or right of the screen. Subjects were informed that each letter can lead to win or to lose money and that the goal was to maximize accumulated money. Note that due to obvious ethical and clinical considerations involving in particular patients with gambling disorder, patients were not rewarded with real money, but with points presented as virtual money. Fast reaction times and behavioral differences between GDP and NGDP indicate that this virtual money was perceived in a way similar to real money. Starting money accumulated was 0 €. The letters B and C were the high-risk (HR) options, leading to a 100€ win 20% of the time and a 70€ loss 80% of the time (Fig. 1*A*). A and D were the low-risk (LR) options leading to a 60€ win 80% of the time and a 30€ loss 20% of the time (Fig. 1*A*). Note that the expected value of LR is +42€, and the expected value of HR is –36€, i.e., in the long-term the LR option leads to an accumulated money increase whereas the HR option leads to an accumulated money decrease. We defined two options with different expected value since we wanted to investigate a defining characteristic of impulse control disorders, i.e., the failure to resist a drive even if it is causing harm to the subject or others (Weintraub et al., 2006; American Psychiatric Association, 2013). In our experimental design this corresponds to the inability to refrain from selecting the high-risk option even if it leads to a loss.

Six different stimulus pairs (A vs. B, A vs. C, A vs. D, B vs. C, B vs. D, C vs. D) were presented. Four of them were conflictual (C), since the subject had to choose between one HR and one LR option: B vs. D, A vs. B, C vs. D, A vs. C. Two were equivalent choice (EC), since the options outcomes were identical: both HR (B vs. C) or both LR (A vs. D). Participants were instructed to choose between the two options, but there was no time restraint, i.e., reaction time was freely chosen. Each choice was followed by two visual feedbacks, the first lasting 1 s, displaying the previous choice outcome (i.e., the money won or lost during the last trial), and the second lasting 1.5 s, indicating the total amount of money accumulated since the beginning of the session. Finally, 0.8 s of black screen preceded the next stimulus presentation. Overall, starting from the second stimulus, each presentation started exactly 3.3 s after the subject response to the previous presentation (see Fig. 1*C*).

The experimenter did not reveal the probability to win associated with each letter; hence the task incorporated a learning phase. Each session was preceded by 12 trials (two for each stimulus pair) for patients to learn the difference between HR and LR. This learning phase duration was previously found to be sufficient for patients to define their strategy (Rosa et al., 2013). After the 12 trials training set, 6 of 6 GDPs showed a preference for the HR option, suggesting that patients learned that the two options were associated with different reward contingencies. Learning phase presentations are not included in behavior or LFP analysis. After the end of the learning phase, two-thirds of the trials (60/90) were conflictual (C) and the rest of the trials were equivalent choice (EC): both HR (15/90) or both LR (15/90). For 1 of 6 GDPs, the session ended earlier after 13 EC LR, 12 EC HR, and 51 conflictual trials. For 1 of 6 NGDPs, 5 conflictual trials were later discarded because of a failure to record reaction time.

### Statistical analysis

Complete description of analysis of subjects’ choices and of processing and analysis of LFP signal preceding options presentation is reported below. Briefly, risk avoidance (probability of choosing LR option in conflictual trials) and reaction times (time interval between options presentation and behavioral response) of GDPs and NGDPs have been compared under different conditions with unpaired Wilcoxon test. Reaction times were then normalized for the average reaction time of each patient and compared across the conditions separately for GDPs and NGDPs with Kruskal–Wallis test corrected for multiple comparisons.

Data processing and part of the statistical analysis was performed in Matlab (Mathworks). Two- and three-way repeated measures tests were performed in SPSS (IBM).

Unless stated otherwise, figures report median value of the variables and interval of confidence of median value, computed as (Chambers et al., 1983)

(1) $$\mathrm{median c}\text{.i.}=\pm \frac{1.57\;\cdot \;\left(\text{75th percentile}-\text{25th percentile}\right)}{\sqrt{\text{samples number}}}.$$

#### Behavioral performance analysis

The behavioral variables collected for each trial during the task were the reaction time (RT), the type of choice (LR, HR), and the money accumulated from the beginning of the task. Risk avoidance (RA) was defined as the fraction of times LR was chosen in conflictual trials (number of LR choices in conflictual trials divided by the number of conflictual trials), and reaction time (RT) as the interval between options presentation and option selection by pressing the corresponding button. Risk avoidance of GDPs (*n* = 6) and NGDPs (*n* = 6) is compared in Fig. 1*B* with paired Wilcoxon test. Risk avoidance of healthy subjects (*n* = 17), GDPs (*n* = 6), and NGDPs (*n* = 6) is compared with Kruskal–Wallis test corrected for multiple comparisons. For the reaction time analysis, we divided the trials in four sets given by the type of trial and the following decision: C LR, EC LR, EC HR, C HR. The number of trials in each set was 139 (C LR), 87 (EC LR), 88 (EC HR), and 212 (C HR) in GDPs and 219 (C LR), 90 (EC LR), 90 (EC HR), and 136 (C HR) in NGDPs. Group–trial type interaction was evaluated with two-way ANOVA in SPSS. GDP and NGDP reaction times were compared overall and for each trial set with Wilcoxon test. Correlation between RT ratio and RA was computed with *corr* function in Matlab.

We computed RA for each subject in the subset of trials in which the accumulated money was above or below the session average, and we compared with a Wilcoxon signed rank test the two RA in GDPs and NGDPs. Interaction between accumulated money and group was evaluated with two-way ANOVA with repeated measures in SPSS. Finally, we measured the extent to which the decisions in conflictual trials (C) in each trial depended by the previous outcome (PO) as follows. If the subjects’ choice was a Bernoulli process, the risk avoidance after each of the four possible POs (the patient chose LR/HR and won/lost) would be independent from the outcome

(2) $$RA_{B}\left(PO\right)=〈RA〉\forall PO.$$

The estimated number of LR choices after each outcome in a memory-less process is then

(3) $$Exp_{B}\left(LR|PO\right)=〈RA〉\;\cdot \;\mathrm{occurrences of PO before C}\text{.}$$

**Figure 3.**
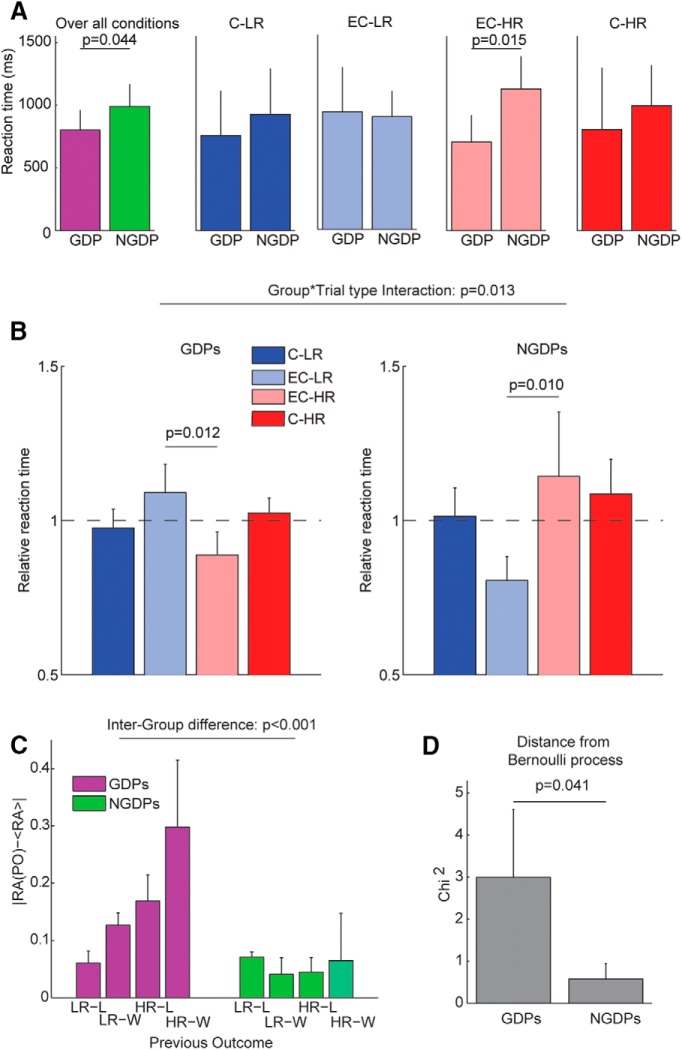
Risk avoidance bias precedes options presentation. ***A***, Comparison of reaction times in GDP (*n* = 6) and NGDP (*n* = 6). From left to right: over all conditions, low-risk choice in conflictual (C-LR) and equivalent choice (EC-LR) trials, high-risk choice in equivalent choice (EC-HR) and conflictual (C-HR) trials. ***B***, Relative reaction times (RT) for the different conditions pooling all sessions of each patient group. The dashed line indicates the median RT. ***C***, Modulation of risk aversion due to previous outcome (PO) for GDPs (purple) and NGDPs (green). ***D***, χ^2^ discrepancy between observed responses and expected responses in an equivalent memoryless Bernoulli process for GDPs (*n* = 6) and NGDPs (*n* = 6). Bars and error bars represent median and median confidence interval, respectively, for all panels. Horizontal line inserts highlight significant differences reporting *p* value (see Results for details) for all panels.

We compared the observed number of LR choices in conflictual trials after each PO with the number expected in case the decisions were memory-less. We used the squared differences between expected and observed value as χ^2^ measure of the goodness of the memory-less fit, i.e. of the extent to which the decisions are independent from previous outcome. In Fig. 3*D*, we compared the χ^2^ between GDPs and NGDPs with Wilcoxon test (*ranksum* function, Matlab).

#### Local field potential (LFP) recording and processing

During the economic task, LFPs were simultaneously captured from the contact pair 0-2 of the DBS electrodes (Fig. 1*D*). Signals were preamplified, differentially amplified (100,000×), and digitized with 1024-Hz sampling rate through the Galileo BE Light EEG amplification system (EBNeuro Spa). Acquired LFPs were preprocessed by applying a 5th-order zero-delay Butterworth bandpass filter in the range [0.5 50] Hz to remove very-low-frequency artifacts and high-frequency noise. A narrow 50-Hz notch filter was also applied to remove electrical noise.

Because we are not looking for inhibition of motion, we did not expect to find any preferred correlation between area of the recording within STN (left or right) and hand motion (ipsilateral and contralateral), but rather a global coordinated inhibition involving both areas. Hence, for the sake of robustness, we averaged the LFP signal coming from the two recording tips (Fig. 1*C*).

Fig. 1*C* displays voltage values of (averaged) LFP recording for single sessions to show absolute values of behavior-dependent fluctuations. However, all the analyses described in the next subsection, involving multiple sessions, were performed on *z*-scored LFPs, to remove the variability associated with the different recording conditions across sessions and focus on the intrasession LFP variations.

#### Analysis of relationship between LFP and behavior

To focus on risk attitude instead of decision-encoding neural activity, we analyzed the LFP recorded in the 3.3 s between the behavioral response to the (n – 1)th presentation and the visual onset of the nth presentation (see Economic decision-making task). We then obtained for each session a set of LFP recording intervals of the same duration.

First we discarded LFPs associated with EC trials (30 trials, see above) as being followed by a forced choice and not useful to understand the relationship between LFP and response. Conflictual choice trials were then divided into (a) C trials followed by choice of the high-risk option LFP_CHR_ and (b) C trials followed by choice of the low-risk option LFP_CLR_. These datasets were the objects of the analysis. For each session, we performed the analyses described below.

#### LFP spectral analysis

Power spectral density was computed with *pwelch* Matlab function over the whole window and over the three functional subintervals (see above). We compared the median power over LR and HR trials for the six subjects of each group with two-sided Wilcoxon rank test (*signrank* function in Matlab). We compared median values across all trials between the six subjects in the GDP and the six subjects in the NGDP group with Kruskal–Wallis test (*kruskalwallis* function in Matlab).

We computed the information about future behavior conveyed by three frequency bands: low-frequency [1 12] Hz, beta [12 30] Hz, and low gamma [30 50] Hz. Spectral information was computed by using as neural signal S (see below) the average log-power of each band in each trial.

#### Low-frequency fluctuations analysis

We analyzed the relationship between the evolution of low-frequency LFP in the interval of interest by applying a low-pass filter at 12 Hz (5th-order Butterworth filter) and computing the average value of the LFP for each of the three intervals (see above) for each trial. We performed on the resulting signal analyses both at the single subject level and comparing all trials of the same group.

##### Subject level analysis

First, we compared for all the subjects in each group for each interval the average value of the LFP preceding HR and LR decisions, to test for significant differences. Then, we evaluated the correlation between LFP value and propensity to risk for each subject, dividing the single-trial average LFPs computed above into four equipopulated percentiles and counting the fraction of trials within each group of LFP that were followed by a low-risk decision (a LFP-averaged risk avoidance). We subtracted from this value the overall risk avoidance of each subject, to see how the LFP modulates risk avoidance. We used these values for two tests. First, we tested (Pearson correlation test, Matlab *corrcoef* function) whether the values of the LFP and the risk avoidance correlated for each interval and condition. Second, we tested whether particularly high values of LFP were associated with a significant discrepancy of risk avoidance from mean value. We compared with a paired Wilcoxon test (*signrank* function Matlab) the average risk avoidance with the LFP-dependent risk avoidance for each LFP percentile.

##### Group level analysis

In a second set of analyses, we grouped the trials for each combination of patient condition (GDP vs. NGDP) and following choice (LR, HR), for a total of 2 conditions × 3 intervals × 2 choices = 12 groups each containing >100 LFP values. We computed the ANOVA three-fold interaction tests for the three factors (with SPSS). As the data were not distributed normally, we computed the test on LFP ranks (Conover and Iman, 1981). As interaction was significant, we computed a second analysis separating LFP value from GPD and NGDP. We computed the interaction for factors intervals and choice with two-way ANOVA with unbalanced design (*anovan* function in Matlab). We computed the significance of the difference of LFP between choices for each condition and interval with a Wilcoxon rank sum test (*ranksum* function in Matlab).

Finally, low-frequency LFP information was computed by using as neural signal S (see below) the average value of LFP over each interval in each trial.

#### Mutual information between LFP and behavior

Mutual information between a set of behaviors B and a set of neural signals S is defined as (Shannon, 1948)

(4) $$I\left(B;S\right)=\underset{s\in S,b\in B}{\sum }P\left(b\right)P\left(s|b\right)\log_{2}\frac{P\left(s|b\right)}{P\left(s\right)},$$

where *P*(*b*) and *P*(*s*) are the absolute probability across all trials of observing a given behavior *b* from the set *B* or given neural signal *s* from set *S*, and *P*(*s*|*b*) is the conditional probability of observing the neural signal *s* in trials in which the (following) behavior is *b*.

Here, we considered as set of behaviors the two possible responses: *B* = [HR, LR]. We computed then the mutual information between this set and different sets of neural signals. First, we considered the average power of the three LFP bands over the whole window of interest (see above), then the average value of the low-passed LFP over each the three different functional intervals (see above). Information was computed with Information BreakDown Toolbox in Matlab (Magri et al., 2009).

We tackled the information bias due to the limited data set (Panzeri et al., 2007) with the following four steps. (1) We grouped together all the trials from all patients from each group to have a sufficiently high number of trials/stimulus. (2) We limited the number of bins of the signal to four (equipopulated) coherently with the binning used in the correlation study (see above), which ensures a conservative but stable measure of information (Ince et al., 2012). (3) We applied the Panzeri–Treves bias correction (Treves and Panzeri, 1995). (4) We compared the resulting values of information with those obtained with 200 bootstrap repetitions (Magri et al., 2009). We considered as significant only values of information having *p* < 0.05 of being generated with a bootstrap procedure, which gives a conservative estimate of information significance (Ince et al., 2012).

#### Dataset limitations

The two groups of patients whose behavior and neural activity we compare in the present work comprise six patients each. This group size is sufficient to obtain statistically significant within- and across-group comparisons, so we performed several analyses considering each subject separately (see Subject level analysis). However, to improve the robustness of our conclusions, we performed a second set of analysis by pooling trials of all subjects from the same group (see Group level analysis).

In reaction time analysis, we compensated for the relatively small sample size by pooling together data from all subjects within the same group after normalizing to the median response time of each subject (Sacré et al., 2016). Average *z*-scored LFPs from all subjects in the same condition were grouped for analysis of variance. Mutual information analysis was computed grouping the normalized neural activity (PSD, *z*-scored LFP) preceding LR or HR decisions of all subjects (Ince et al., 2012) of each group (see Methods for details). Note that these analyses were complemented by the subjectwise analysis of the LFP PSD and the correlation between LFP and risk avoidance.

## Results

The patients were asked to perform a two-alternative forced-choice task choosing between two letters presented on a screen (see Methods and Table 1 for details). The letters were associated with a probabilistic economic outcome (Fig. 1*A* and Methods). The HR option (letters B and C) had a high maximum reward (100 €) associated with a low probability (20%) and a negative expected value (–36 €); the LR option (letters A and D) had a lower maximum reward (60 €) associated with a high probability (80%), and a positive expected value (+42 €; Rosa et al., 2013; Fumagalli et al., 2015). Each session consisted of 90 trials, preceded by a short learning phase (see Methods). Two-thirds of the trials were conflictual (C), i.e., the subject had to choose between HR and LR. The others were equivalent choices (ECs), i.e., both letters were associated with either HR or LR. The choice outcome and the total amount of money accumulated from the beginning of the session were displayed on the screen (Fig. 1*C*, top) during the 3.3-s interval between each option selection and the following option presentation.

The patients performed the task 4 d after DBS surgery, when the extensions connected to the extracranial part of the STN lead were accessible for LFP recordings (see Methods, Fig. 1*D*, Fig. 2, and Table 2 for details). The analysis focused on the interval that preceded options presentation (Fig. 1*C*, bottom, and Methods) to identify the features of the STN LFP signal correlated to the behavioral bias given by the attitude toward risk (Sacré et al., 2016). The selected interval also ensures that STN activity was not motion related.

## Decision bias precedes options presentation

Gambling disorder, like all impulse control disorders, is characterized by a difficulty in resisting a drive even if it leads to a personal loss (Weintraub et al., 2006; American Psychiatric Association, 2013). In our case, this corresponds to preference toward the HR option even if the expected value is negative. We characterized for each patient the ability to resist this drive and select the most convenient option by means of risk avoidance (RA), measured as the fraction of times LR was chosen on conflictual trials. RA was significantly lower for GDPs than for NGDPs (intermedian difference [IMD] = –0.16; Wilcoxon test [WT], *p* = 0.013, Fig. 1*B*), and all GDPs showed a preference for the HR option (RA < 0.5 for 6/6 GDPs, sign test *p* = 0.031). This finding was consistent with behavioral screenings acquired before the recording sessions (see Methods). Although only 4 of 6 NGDPs selected a low-risk strategy (RA > 0.5) over the whole task, the risk avoidance of NGDPs and a control group of healthy subjects (see Methods) did not differ significantly (IMD = –0.058; WT, *p* = 0.99).

We compared for the two patient groups the reaction time (RT) of each decision, i.e., the interval between the options presentation and the response (Fig. 3*A*). As expected (Napier et al., 2015), the RTs of GDPs were overall faster compared with those of NGDPs (IMD = –188 ms; WT, *p* = 0.044). However, when we took into account both group and trial type in determining the RTs, we found a significant interaction between the two factors (two-way mixed ANOVA *F*(3,1052) = 3.59, *p* = 0.013). Note that significance of interaction holds without subject pooling (two-way mixed ANOVA with repeated measure on ranks *F*(3,30) = 3.847, *p* = 0.019). Conflictual and equivalent choice RTs (neglecting the response type) were not significantly different in GDP (KW test, *p* = 0.55) or NGDP (KW test, *p* = 0.21). RTs were then analyzed separately for each patient group, taking into account trial type and response. GDPs had faster RTs than NGDPs only on trials in which two HR options were presented (EC-HR trials; IMD = –423 ms; WT, *p* = 0.015). During trials in which two LR options were presented (EC-LR), GDPs were actually slower, although not significantly (IMD = 37 ms, WT, *p* = 0.94). Hence, the tendency of GDPs to make decisions more quickly than NGDPs strongly depended on the options presented.

The relative RTs (normalized to the median RT of each subject) across trial type for each patients group were then compared to understand how RTs were modulated by the trial type. Reaction times across trial types were significantly different for GDPs (Fig. 3*B*, left, KW test with Tukey–Kramer correction for multiple comparisons [KWMC], *p* = 0.0035). *Post hoc* analysis revealed that the relative RTs of GDPs were significantly shorter on EC-HR trials than on EC-LR trials (IMD = –0.20; *p* = 0.012). Also, for NGDPs, reaction times across trial types were significantly different (Fig. 3B, right, KWMC, *p* = 0.0066). *Post hoc* analysis revealed that for NGDPs, the relative RTs on EC-HR trials were significantly longer than on EC-LR trials (IMD = +0.35; KWMC, *p* = 0.01). In other words, GDP reactions were slower when presented with two LR options, whereas NGDP reactions were slower when presented with two HR options, even if in both cases there was no decision to be made. These findings are compatible with a decision bias occurring before options presentation (usually favoring LR for NGDPs and HR for GDPs). RTs on EC trials were slower when the preferred option was not available, requiring subjects to switch their decision strategy. Consistent with these findings, the ratio of RTs on EC-HR and EC-LR trials sets strongly correlated with risk avoidance across both GDPs and NGDPs (*R* = 0.89, Pearson correlation test [PCT], *p* = 0.0001). A similar correlation was also observed in healthy subjects (*R* = 0.53, PCT, *p* = 0.028). These results suggest that both GDPs and NGDPs had a strong decision bias before the options were presented and that RTs depended largely on the agreement between the planned response and the options available.

As expected, neither GDPs nor NGDPs behaved deterministically in conflictual trials, as each subject took a specific decision on each single trial. We examined then whether the single-trial decision was more affected by a global evaluation of the strategy or by a reaction to recent decision/outcome history. One possible global strategy would be that subjects modulate their risk attitude according to money accumulated from the beginning of the session, for instance according to a saturating utility curve (Bernoulli, 1954). The relationship between RA and accumulated money was significantly different in the two groups (two-way mixed ANOVA with repeated measures *F*(1,10) = 5.69, *p* = 0.0382). The RA of NGDPs was significantly lower when the accumulated money was lower than session average (IMD = –0.13; paired WT, *p* = 0.031). For 2 of 6 NGDPs, the increase in the risky behavior associated with low accumulated money was so strong (ΔRA = –0.32 and –0.23) to lead to an overall risky strategy (RA < 0.5).The accumulated money did not exert instead any impact on GDP RA (IMD = 0.0097; paired WT, *p* = 0.81). We examined then whether RA was specifically influenced by the outcome of the decision taken in the preceding trial (LR/HR followed by loss or win): we computed for each group the discrepancy between the overall RA and RA given the previous outcome (PO). Two-way ANOVA indicated that the discrepancy was different between the two groups (*F*(1,40) = 14.4, *p* < 0.001) and for different POs (*F*(3,40) = 7.3, *p* = 0.001) with a significant interaction between the two factors (*F*(3,40) = 3.2, *p* = 0.034), as is shown in Fig. 3*C*. We measured then for each subject how consistently the sequence of decisions in conflictual trials could be approximated by a Bernoulli process in which each choice is independent (see Methods). The influence of the previous outcome on RA was significantly higher in GDPs than in NGDPs (χ^2^ distance from Bernoulli process, IMD = 2.42, WT, *p* = 0.041, Fig. 3*D*). This indicates that GDPs decisions were significantly more influenced than those of NGDPs by the preceding decision’s outcome. Overall, these results define an interval between the display of the previous decision consequence and the onset of the following trial where neural activity could affect the risk attitude of GDP patients.

## Bandwise STN LFP spectral content correlates with patient condition, but not with risk avoidance

**Figure 4.**
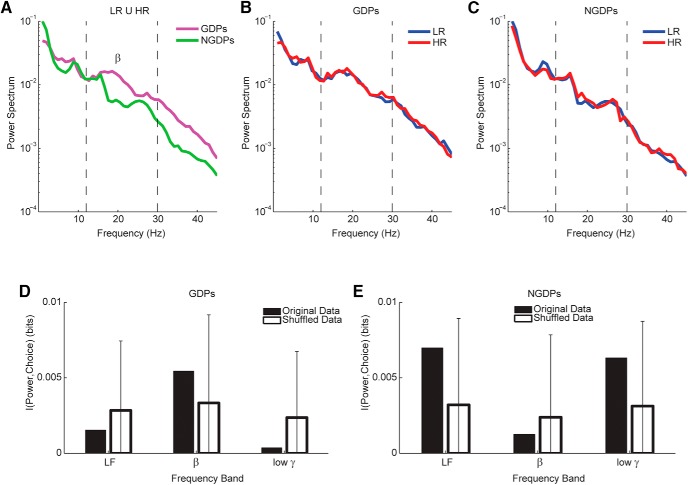
Spectral modulations of subthalamic nucleus local field potential and future decisions. ***A***, Average power spectrum of STN LFP preceding options presentations over all trials for patients with gambling disorder (GDPs) and without (NGDPs). Dashed lines indicate beta band (12–30 Hz). ***B***, Average power spectrum of STN LFP preceding options presentations over all low-risk (LR) and high-risk (HR) tasks for patients with gambling disorder (GDPs). ***C***, Same as ***B*** for patients without gambling disorder (NGDPs). ***D***, Mutual information between future choice and average power of the different bands for GDPs. White bars and error bars, respectively, represent median and 75th percentile of bootstrap information (see Methods). ***E***, Same as ***D*** for NGDPs.

We first analyzed the spectral content of STN LFP recorded in the whole interval for GDPs and NGDPs. The two groups did display significant differences in spectral content over the whole session (KW test, *p* = 0.25). The peak of the relative difference in power between the two groups was found at 19 Hz (relative difference in power = 204%), with a striking resemblance with the spectral difference between PD patients with and without impulse control disorder found in Rodriguez-Oroz et al. (2011) (Fig. 4*A*).

We compared then for each group separately the power spectra in intervals preceding HR and LR choices in conflictual trials (Fig. 4*B*, *C*). The spectra did not show significant differences (KW test *p* > 0.5 for both groups). The peaks of the relative difference in power between the two conditions were found below beta band and were much smaller than in the previous comparison (28% at 6 Hz for GDPs and 27% at 9 Hz for NGDPs). The window of interest was divided into three intervals, characterized by different screen display (Fig. 1*C*, top). Different intervals were likely to be associated with different neural activity. We wondered then whether the spectra in the three intervals were different for different future choices in the two groups. This was not the case, as there was no significant choice × interval interaction (two-way ANOVA with repeated measures, *F*(2,30) = 0.27, *p* = 0.7 for NGDPs and *F*(2,30) = 1.2, *p* = 0.3 for GDPs).

These results suggest that the overall STN LFP spectrum did not correlate with future choice. To further corroborate this conclusion, we computed the amount of information about future decision carried trialwise by the average power of the beta band ([12 30] Hz), the low frequencies below beta ([1 12] Hz), and the gamma-range frequencies above beta ([30 50] Hz; see Methods). We found that no band carried significant information about future choice in GDPs or NGDPs (Fig. 4*D*, *E*, *p* > 0.05, bootstrap test).

## STN low-frequency fluctuations correlate with risk avoidance in GDPs, but not in NGDPs

That the power of a neural signal does not carry information about a given behavioral feature does not imply that the signal is not informative, as information might be encoded in the signal phase, e.g., in the timing of the fluctuations of the signal relative to the behavioral time frame. Indeed, although bandwise spectral analysis did not capture significant correlations between STN LFP and risk avoidance, we investigated whether the low-pass-filtered LFP of GDPs in the interval of interest was different when preceding conflictual trials ending with an HR or LR decision. Note that fluctuations of LFP bandpassed in beta and above had interval averages close to zero.

**Figure 5.**
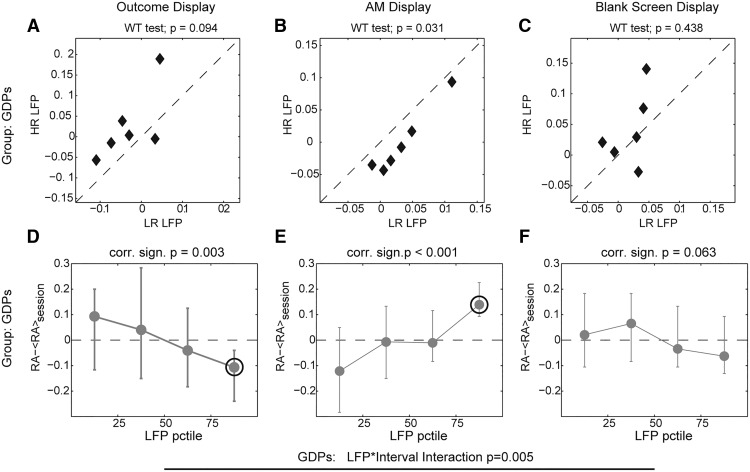
Subjectwise correlation between low-frequency STN LFP preceding options presentation and risk avoidance in GDPs. ***A–C***, Comparison of the average value of GDPs LFP in the three intervals for the two conditions. Title reports significance of Wilcoxon rank test. ***D–F***, Modulation from average risk avoidance associated with trials in which LFP averaged over the three different intervals belongs to the four percentiles. Markers and bars indicate medians and range over GDP. Circle indicates significant difference from average value (*p* < 0.05, Wilcoxon rank test).

When analyzing the LFP subjectwise, we found a significant choice × interval × group interaction (three-way mixed ANOVA with repeated measures, *F*(6,60) = 2.31, *p* = 0.046); hence, we analyzed the two groups separately. In GDPs, we found a significant choice × interval interaction (two-way mixed ANOVA with repeated measures, *F*(6,30) = 3.894, *p* = 0.005), so we analyzed each interval separately. We compared the median low-frequency LFP preceding HR and LR choices in the three intervals for each GDP (Fig. 5*A–C*). We found that LFP tended to be higher for HR (Wilcoxon rank test (WRT), *p* = 0.094) in the first interval, while was significantly higher for LR in the second (WRT, *p* = 0.031) and displayed no differences in the third interval (WRT, *p* = 0.438). We wondered then whether the LFP activity in the different intervals correlated subjectwise with changes in risk avoidance (Fig. 5*D–F*). We found that risk avoidance was significantly anticorrelated with LFP activity in the first interval (*R* = –0.57, PCT, *p* = 0.0035) and significantly correlated with LFP activity in the second interval (*R* = 0.70, PCT, *p* = 0.0001), whereas we found no correlation between LFP in the third interval and behavior (*R* = –0.38, PCT, *p* = 0.063), in agreement with results in Fig. 5*A–C*. In particular, for 6 of 6 subjects, trials with LFP in the 75th percentile in the first/second interval were associated with a decrease/increase in risk avoidance (WRT, *p* = 0.0313 for both intervals, Fig. 5*D*, *E*).

**Figure 6.**
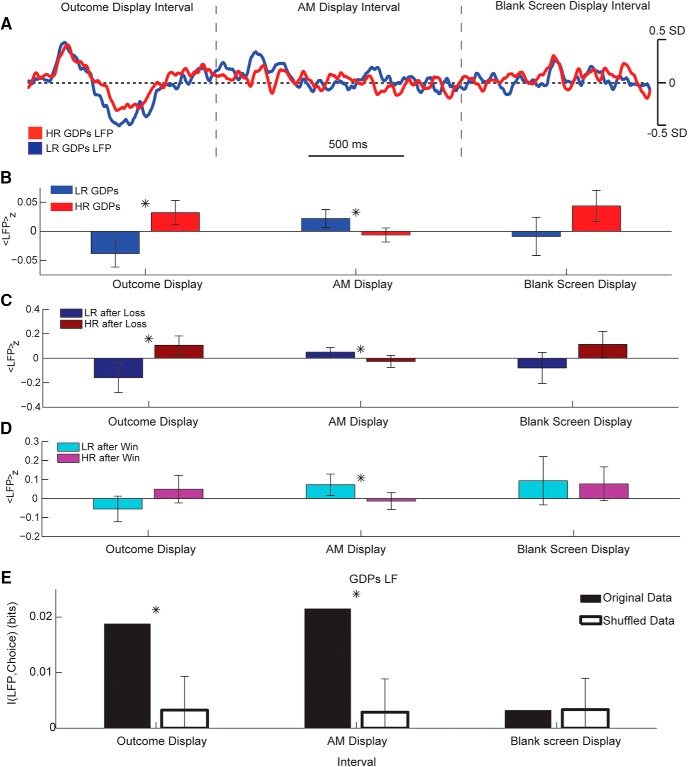
Groupwise correlation between low-frequency STN LFP preceding options presentation and risk avoidance in GDPs. ***A***, Grand average of *z*-scored LFP preceding task in which GDPs opted for HR (red) or LR (blue) option. Areas indicate median value confidence. Horizontal dotted line represents *z* = 0. Vertical dashed lines indicate the interval in which the outcome of previous choice, the accumulated money, and the blank screen are displayed. See Fig. 1*C* for details. ***B***, Average value of GDPs LFP in the three intervals for the two conditions. Markers indicate *p* < 0.05 significant difference. ***C***, Same as B, considering only trials in which the outcome of the previous choice was a loss. ***D***, Same as B, considering only trials in which the outcome of the previous choice was a win. ***E***, Mutual information between LFP levels in the different intervals and future choice (black bars). White bars and associated error indicate average and 75th percentile bootstrap information over 200 permutations (see Methods). Marker indicates *p* < 0.05 significance of information (bootstrap test with Bonferroni correction).

To perform further analysis overcoming the limited number of subjects available, we grouped then all the trial-averaged LFPs for the two groups (Fig. 6*A*). When considering group data, GDPs and NGDPs displayed a different level of activity across the different intervals (significant group × choice × interval interaction, *F*(2,2106) = 5.82, *p* = 0.003, three-way ANOVA on ranks) and hence were analyzed separately. In the GDP group, we found a significant interaction of choice × interval for the LFP average value over the three intervals (two-way ANOVA, *F*(2,1047) = 3.55, *p* = 0.029), then we analyzed each interval separately (Fig. 6*B*). The average LFP in the first interval was considerably lower in trials preceding LR decisions (WRT, *p* = 0.0024), whereas the opposite was true for the second interval (WRT, *p* = 0.0012), and no decision-related difference was found in the third interval (WRT, *p* = 0.12). This indicates that in GDPs, HR and LR decisions are associated with significantly different pattern of LFP activity before options presentation. Because the two intervals in which we found significant differences were associated with the presentation of the results of the previous decision, we performed for each interval a two-way analysis taking into account the factors “future decision” and “outcome of previous decision.” We found that there was no significant interaction between the two factors (*F*(1347) < 0.2, *p* > 0.7 for the first two intervals, *F*(1347) = 3.4, *p* = 0.065 for the last interval). Indeed, when considering only trials after a loss (Fig. 6*C*), the LFPs were significantly different for the first two intervals (WRT, *p* = 0.016 and *p* = 0.027, respectively) and tended to be different in the third one (WRT, *p* = 0.084). When considering only trials after a win (Fig. 6*D*), the LFPs were significantly different for the second interval (WRT; *p* = 0.023) and tended to be different in the first one (WRT; *p* = 0.070). No significant difference was found between LFPs preceding the same future choice but following different previous outcomes (WRT, *p* > 0.1 for all intervals and both future choices). We can consider then the difference between the LFPs associated with different future decisions to be largely independent from the previous outcome. Finally, we computed the mutual information (see Methods) between LFP activity in the different intervals preceding options presentation and the ensuing selected option (Fig. 6*E*). We found that LFP in the first two intervals carried significant information (*p* < 0.05, bootstrap test with Bonferroni correction) about future choices.

These results shows that in GDPs, low-frequency fluctuations in STN LFP preceding option presentations are correlated with risk avoidance in the next trial, suggesting that STN activity might play a role in determining the risk bias for this group. In other words, the STN carries information about the ability of GDPs to choose in the future a safe option against their general bias toward risk, suggesting that STN might be involved in this behavioral suppression, as observed in different behavioral tasks (Jahanshahi et al., 2015b). Note that STN LF LFP in GDPs did correlate with future choice, but, for a given choice, did not correlate with reaction time (Pearson correlation test, *p* > 0.2 for every condition and interval), in accord with results reported in Zénon et al. (2016), suggesting that STN LF LFP correlated with reward evaluation rather than with conflict.

**Figure 7.**
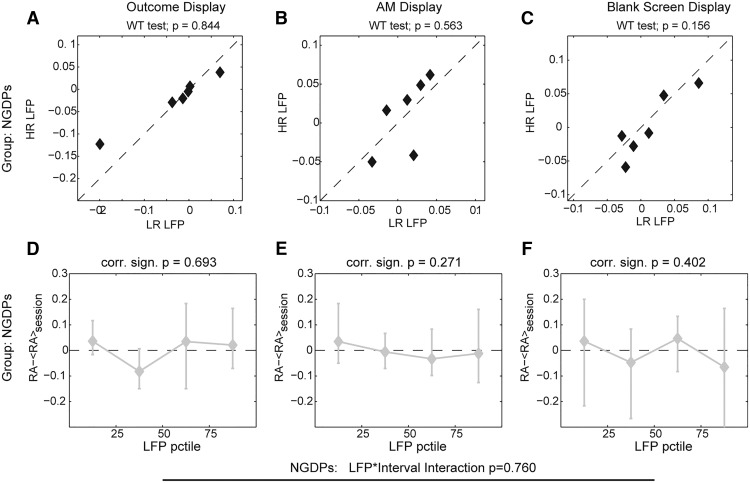
Subjectwise correlation between low-frequency STN LFP preceding options presentation and risk avoidance in NGDPs. ***A–C***, Comparison of the average value of NGDPs LFP in the three intervals for the two conditions. Title reports significance of Wilcoxon rank test. ***D–F***, Modulation from average risk avoidance associated with trials in which LFP averaged over the three different intervals belongs to the four percentiles. Markers and bars indicate medians and range over NGDP.

**Figure 8.**
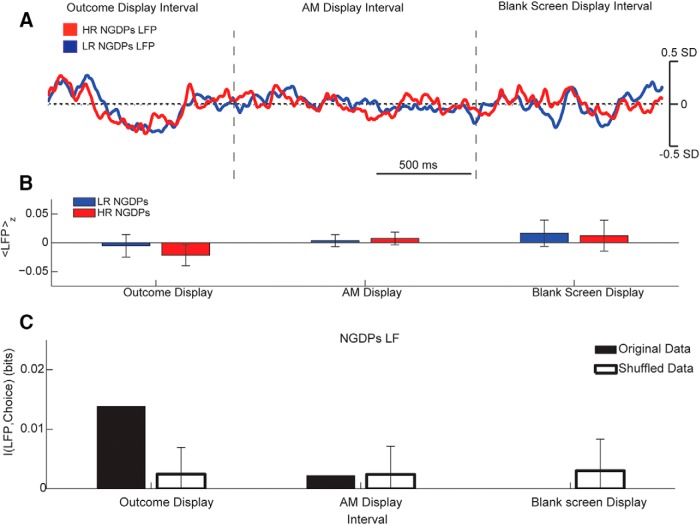
Groupwise correlation between low-frequency STN LFP preceding options presentation and risk avoidance in NGDPs. ***A***, Grand average of *z*-scored LFP preceding task in which NGDPs opted for HR (red) or LR (blue) option. Areas indicate median value confidence. Horizontal dotted line represents *z* = 0. Vertical dashed lines indicate the interval in which the outcome of previous choice, the accumulated money, and the blank screen are displayed. See Fig. 1*C* for details. ***B***, Average value of NGDPs LFP in the three intervals for the two conditions. ***C***, Mutual information between LFP levels in the different intervals and future choice (black bars). White bars and associated error indicate average and 75th percentile bootstrap information over 200 permutations (see Methods).

We repeated for NGDPs the same subjectwise analysis performed for GDPs, and we found no LFP × interval interaction (two-way ANOVA repeated measures, *F*(6,30) = 0.558, *p* = 0.76). Indeed, we did not find any difference between LFP preceding HR or LR (WRT, *p* > 0.15 for all intervals, Fig. 7*A–C*) or LFP-RA significant correlation (|*R*| < 0.25, PCT, *p* > 0.2 for all intervals; Fig. 7, *D–F*) for any interval. The low-frequency LFPs in NGDPs were relatively unrelated to the following decisions (Fig. 8*A*). We found no significant interaction of choice × interval for the LFP average value over the three intervals (two-way ANOVA, *F*(2,1059) = 0.07, *p* = 0.93), and in no interval did we find significantly different LFPs associated with the future decisions (Fig. 8*B*, WRT, *p* > 0.3 for all intervals). Note that NGDP LF LFP did not correlate to reaction times either (*p* > 0.05 for all intervals and conditions). Finally, in no interval did low-frequency LFP of NGDPs STN carry significant information about future choices (*p* > 0.05 bootstrap test with Bonferroni correction, Fig. 8*C*).

The stronger correlation between behavior and STN activity before option presentation observed in GDPs compared with NGDPs is in agreement with the fact that previous outcomes contribute much more in GDP decisions (Fig. 3*D*). Moreover, the lack of significant correlation between STN activation and risk avoidance in NGDPs supports the hypothesis that the STN plays a crucial role in suppressing unsafe urges (Aron, 2011). This suppression might then be present only in GDPs who have an unsafe urge to take risks but not in NGDPs who spontaneously lean toward a safer choice.

## Discussion

We compared behavior and STN neural activity of Parkinson’s disease patients with and without gambling disorder during an economic decision-making task. The main differences in the behavioral responses were related to reaction times and structure of decision-making. First, the longest/shorter reaction times for NGDPs were for equivalent-choice trials in which the options were both HR or LR, whereas the opposite was true for GDPs. This suggested the possibility that the choice was strongly biased before option presentations and that patients needed time to change strategy when the preselected option was not available. Second, we found that GDP decisions were strongly affected by the outcome of the immediately preceding trial, whereas this was not true for NGDPs. This indicated that GDP decisions were determined in the interval between two consecutive trials.

The results of our analysis of STN activity demonstrated that, when a subject affected by gambling disorder faces economic choices, the STN activity preceding options presentations correlates with the ability to select the low-risk option (with a larger expected value) despite the overall preference toward risky options. We argue that this suggests that the STN plays a role in determining upcoming economic decisions by opposing pathologic risk propensities. The seminal paper by Frank et al. (2007) showed that STN sends a “global no-go” signal that “temporarily prevents the execution of any response” in the “face of conflict,” after the options have been presented. This behavioral phenomenon is referred to as “reactive global stopping” (Aron, 2011; Jahanshahi et al., 2015b). The results presented here are compatible with the hypothesis that the STN might serve also as a “proactive selective control” (Aron, 2011), i.e., a complementary function that prepares to stop a selected response tendency in an upcoming task. In other words, our results support the idea that the role of STN goes beyond putting decisions on hold after a conflict is detected, but includes suppressing an undesired behavior after an internal bias toward an unfavorable action is detected.

We have in particular found future decisions to be correlated with interval-dependent STN LFP fluctuations in the low frequencies (LF, <12 Hz) below the beta band ([12 30] Hz). These two frequency bands have different functional properties in STN (Jahanshahi et al., 2015a). In particular, LF and beta band in STN LFP have been linked to different aspects of decision-making (Rodriguez-Oroz et al., 2011), with LF being primarily associated with reward level (Zénon et al., 2016) and risk (Rosa et al., 2013), whereas beta is primarily associated with conflict (Brittain et al., 2012). A recent work links low-frequency STN activity with the “level of cautiousness” of subjects presented with an ambiguous perceptual discrimination (Herz et al., 2016). This is coherent with results establishing a specific functional link between low-frequency STN activity and the medial prefrontal cortex, whereas beta band correlates with motor cortex (Rodriguez-Oroz et al., 2011; Herz et al., 2017; Horn et al., 2017). Although the motor role of STN is usually associated with beta band, cognitive functions have been found to be related to different frequencies below the beta band. To keep our results as general as possible and avoid frequency band hand-picking, we considered everything below the beta band as low frequency. Our results support the view of such low frequencies being related in STN to cognitive functions.

We have here observed a significant relationship between STN activity and future decisions. The first limitation of this finding is that we do not have a mechanistic explanation for this finding. One hypothesis might be that an outcome inducing a decrease in the risk drive triggers an activation of the STN, which we observe as a large low-frequency deflection in the LFP, followed by a decrease in activity, which we observe as a slow rebound in the LFP. This hypothesis could be tested by modulating the different intervals of the task. The second limitation is that correlation obviously does not imply causality. A direct test of causality, and not mere correlation, between STN and future behavior would be to properly stimulate the STN of GDPs in the interval between economic risk trials and observe the expected reduction in risky behavior. Such a test would also be the first step toward an electroceutical therapy for gambling disorder.

The cognitive role of STN may generalize to individuals without PD. In fact, the inhibitory role of STN in decision-making seems to be qualitatively similar for PD patients and healthy subjects (Frank et al., 2007), because it is probably not affected by the neurologic disease or dopamine medication (only by DBS, which was off in our study). Our results support the hypothesis put forth by Jahanshahi et al. (2015b) that the STN contributes to proactive inhibition via its functional connections through the striatum (Majid et al., 2013; known to be involved in impulsivity Buckholtz et al., 2010) to the prefrontal cortex (Cavanagh et al., 2011; Rodriguez-Oroz et al., 2011), an area strongly related to human decision-making in the face of uncertain outcomes (Bechara et al., 1994; Domenech and Koechlin, 2015). Our findings suggest that STN might take part in proactive selective inhibition by suppressing the impulsive attraction of GDPs for the risk associated with high but unlikely rewards and favor a rational preference for options associated with positive expected value. This interpretation also accounts for the lack of influence of STN in NGDPs, because risk propensity is missing or weaker in NGDPs and hence no suppression is needed. Our results and this interpretation are coherent with the results in an identical task presented in Rosa et al. (2013), in which low-frequency STN LFP modulation were associated with conflictual stimuli in GDPs but not NGDPs. Additionally, the role of STN in high-conflict tasks (Frank et al., 2007) and difficult moral decisions (Fumagalli et al., 2011; Fumagalli and Priori, 2012) can be interpreted within this framework. The lack of proactive selective inhibition might underlie most impulse control disorders, which indeed show a high rate of comorbidity (Weintraub et al., 2015), and might have overlapping neural mechanisms (Averbeck et al., 2014). The STN may then play a role in other impulse-control disorders. Our findings about the relationship between risky decisions and STN activity in GDPs lay the groundwork for innovative pharmacological and neuromodulatory strategies that target the STN to efficiently tackle addiction and impulse control disorders.

## Synthesis

Reviewing Editor: Bradley Postle, University of Wisconsin Decisions are customarily a result of the Reviewing Editor and the peer reviewers coming together and discussing their recommendations until a consensus is reached. When revisions are invited, a fact-based synthesis statement explaining their decision and outlining what is needed to prepare a revision will be listed below. Note: If this manuscript was transferred from JNeurosci and a decision was made to accept the manuscript without peer review, a brief statement to this effect will instead be what is listed below.

I've looked over your responses to the reviews from your J. Neurosci. submission, as well as to the manuscript that you have submitted to eNeuro, which was revised accordingly. I judge your revisions to have been satisfactory to make your submission suitable for publication without needing to go out for peer review.
